# Parameters of prediction: Multidimensional characterization of top-down influence in visual perception

**DOI:** 10.1016/j.neubiorev.2023.105369

**Published:** 2023-08-22

**Authors:** Javier Ortiz-Tudela, Victoria I. Nicholls, Alex Clarke

**Affiliations:** a Department of Psychology, Goethe University Frankfurt, Germany; b Mind, Brain, and Behavior Research Center, Department of Experimental Psychology, University of Granada, Spain; c Department of Psychology, University of Cambridge, UK

**Keywords:** Visual perception, Memory, Prediction, Top-down, Expectations

## Abstract

Despite the recent popularity of predictive processing models of brain function, the term *prediction* is often instantiated very differently across studies. These differences in definition can substantially change the type of cognitive or neural operation hypothesised and thus have critical implications for the corresponding behavioural and neural correlates during visual perception. Here, we propose a five-dimensional scheme to characterise different parameters of prediction. Namely, *flow of information*, *mnemonic origin*, *specificity*, *complexity*, *and temporal precision*. We describe these dimensions and provide examples of their application to previous work. Such a characterisation not only facilitates the integration of findings across studies, but also helps stimulate new research questions.

## 1 Introduction

Over the last few decades, the conceptualisation of brain function in terms of predictive processing has gained considerable momentum. Despite its early roots more than a century ago, cognitive accounts that consider the dynamic interaction between incoming sensory information and prior knowledge are now being regarded as powerful frameworks to explain brain function. At their core, all predictive processing accounts rest on the notion that top-down predictions are contrasted with bottom-up inputs, which can then be used to improve future predictions. From the study of dendritic communication to models of social interactions, predictive processing has the potential to explain human cognition and behaviour across very different hierarchical levels.

**Fig. 1:**
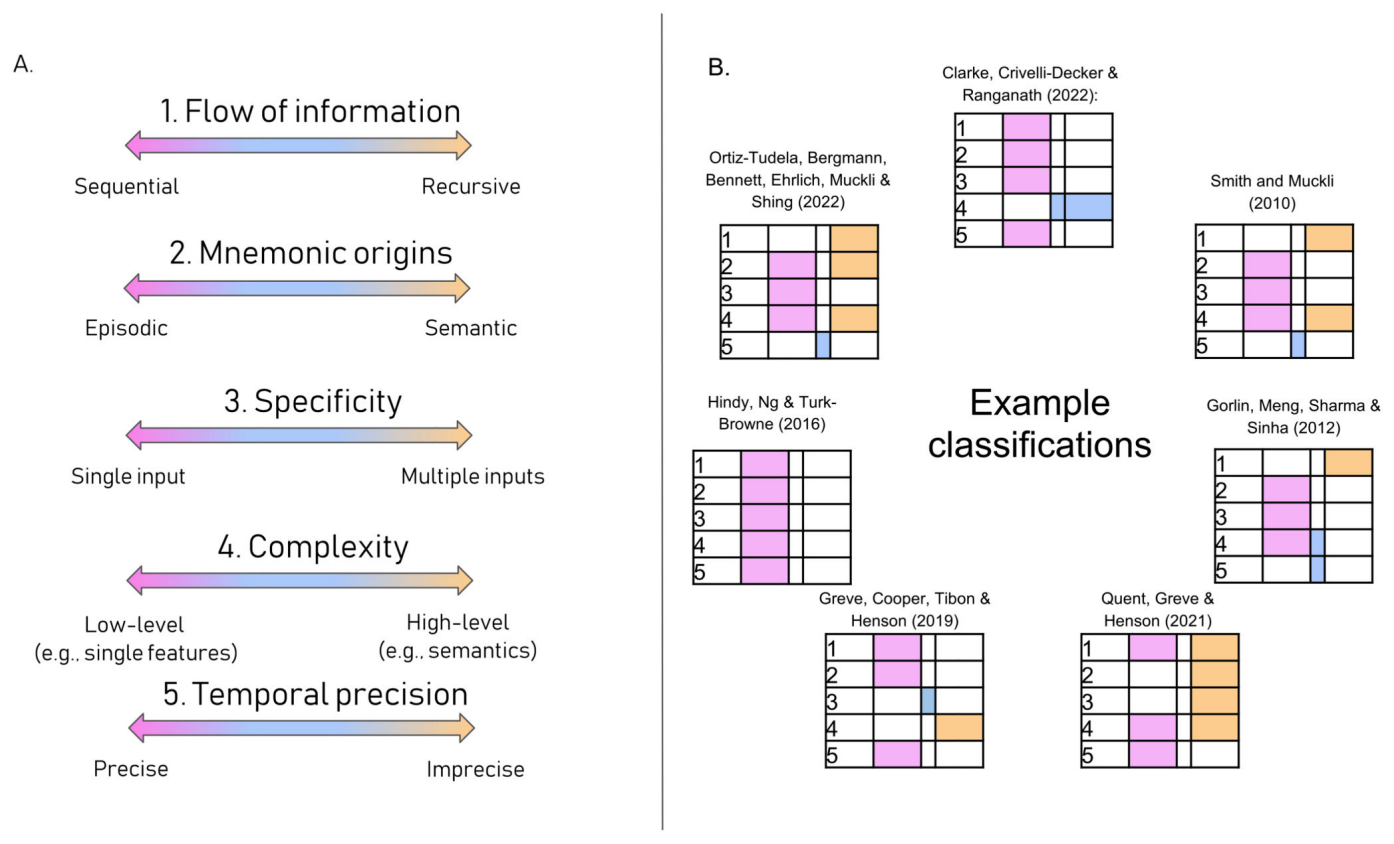
Multidimensional characterisation. A) The parameters that can influence prediction during visual perception and B) how such parameters map onto specific experiments.

However, the operationalisation of the concept of “prediction” is instantiated in different ways to match the respective field of study or experimental paradigm. Some of these instantiations deviate substantially from each other which can severely harm attempts at integrating insights from different studies. In this opinion piece, we argue that a careful characterisation of the type of predictive processing evoked by each study is necessary. Such characterisation would be highly beneficial, especially when searching for neural and behavioural correlates of predictions. To this end, we identified at least five non-orthogonal dimensions along which different types of predictions can be described, incorporating factors like the nature of the information carried by the predictions, and how and when predictions are initiated during visual perception (Fig. 1).

## 2 Multidimensional characterisation

### 2.1 Flow of information

Consider a train travelling along a track. Here we have information about the speed and direction of motion, with the content of our predictions relating to the moment-to-moment sensory changes our visual system will experience. This provides an example of recursive predictions (sometimes known as perceptual predictions) which are implicit, obligatory, and constant, thus turning our perceptual experience into a recursive interaction between predictions and inputs. Despite the input dynamically changing, the time lag between these elements is treated as negligible in practice (Ortiz-Tudela et al., 2023; Smith and Muckli, 2010), creating an ongoing loop between predictions and perceptions. In contrast, we can also anticipate the presence of some new, distinct information that is not present in the current perceptual experience (sometimes known as mnemonic predictions). One example of such sequential predictions would be those evoked through learned cue-item relationships (e.g., Kok et al., 2014), where predictions have a concrete onset triggered by the cue, and a time lag is assumed to pass until the expected item appears. The cue-item predictive relationship can be generalised to sequences, where each item predicts the next, but still with an assumed time delay (e.g., Clarke et al., 2022). Rather than implicit and continuous, sequential predictive processes are explicit and finite.

### 2.2 Mnemonic origins

Predictions are drawn from previous experience, and therefore, the nature of that mnemonic information will impact the information conveyed by predictions, as well as the neural substrates engaged in their generation (Ortiz-Tudela et al., 2023). One salient distinction regarding mnemonic content is between semantic and episodic information. On one hand, memory-guided predictions can reflect more episodic-like information, being linked to a specific spatiotemporal context and with the source likely being the medial temporal lobe and parietal cortex (Clarke et al., 2022; Gunseli and Aly, 2020; Hindy et al., 2016; Kok et al., 2014; van Kesteren et al., 2012). Such predictions may provide perceptually rich information as they relate to real experienced situations. On the other hand, predictions related to our general semantic knowledge of the world may be more abstract and gist-like (van Kesteren et al., 2012) thus depending on ventral temporal and prefrontal regions (Gorlin et al., 2012; Ortiz-Tudela et al., 2023). In between these two extremes, we would find predictions based on neither semantic nor episodic information. One example would be predictions based on lower-level statistical regularities such as co-occurrences of perceptual features, which might still provide perceptually rich information. For example, predicting that a straight line will continue to be straight once a partially occluding object disappears. Such predictions are based on learned statistical regularities, but without a clear episodic or semantic origin.

### 2.3 Specificity

Predictions can also differ in terms of whether they relate to one specific expected input, for example, a specific image of a specific tiger, whether they reflect a collection of expected inputs, for example, any tiger but not a specific image, or cases where predictions are weak but corresponding to a large number of potential expected inputs. Statistical learning paradigms often require participants to learn to expect a specific stimulus following a cue (Clarke et al., 2022; Hindy et al., 2016; Kok et al., 2014); conversely, entering a kitchen will activate a large range of semantically congruent concepts we might expect to encounter (Quent et al., 2021; van Kesteren et al., 2012). This dimension is reflected in Bayesian predictive accounts through the distribution of priors. At one end of the dimension, predicting a single, known input, will be characterised as a precise (i.e., sharp) prior. If multiple inputs are predicted (e.g., three possible orientations of visual gratings are equally likely), this will result in a more imprecise prior, represented at an intermediate position on the dimension. When there are a large number of equally predictable candidates, this would result in a more flat prior and constitute the other end of the dimension. This dimension has parallels with contextual priors discussed by Quent et al. (2021), within the PIMMS framework (Henson and Gagnepain, 2010) which further outlines the potential implications of different priors for declarative memory processes.

### 2.4 Complexity of reactivated information

Predictions are used by the brain to explain (away) inputs which are intrinsically noisy in a process known as disambiguation. Such disambiguation can occur at different levels of the processing hierarchy, indicating that the information that is useful for predictive processing can be relatively low or high level. These different levels of information fed-back by predictions will relate to the precise cortical site of reactivation (Henson and Gagnepain, 2010; Clarke et al., 2022; Hindy et al., 2016). For instance, predictions concerning low-level information such as the orientation of visual gratings are likely to be represented in primary visual cortex rather than higher-level visual regions. Conversely, predictions about object information are more likely to be represented in higher levels of the visual hierarchy (Gorlin et al., 2012; Ortiz-Tudela et al., 2023).

### 2.5 Temporal precision

Predictions might not only relate to the anticipated content but can also provide temporal information about the onset of that content. Predictions relating to precise temporal information can be important for enabling efficient actions, such as when playing tennis (recursive prediction, perceptual), and experimental paradigms involving navigation or sequential stimuli (sequential predictions, episodic) with specific temporal dynamics (Clarke et al., 2022; Hindy et al., 2016; Kok et al., 2014). In contrast, predictions can also signal that something specific will happen in the future but provide imprecise information about when. For example, going to a restaurant we might have a specific expectation that we will see a menu, but we will have imprecise information about the timing. The neural mechanism driving precise temporal predictions can also be very different. For example, while the hippocampus might be particularly important for representing anticipated future states with temporal precision (Eichenbaum, 2017), this same mechanism would not be expected to support temporally precise recursive predictions.

## 3 Conclusion

The broad use of prediction across different studies creates serious problems when aggregating findings to build new theories, or when assessing empirical support for existing ones. Here we propose a five-dimensional scheme to help circumvent these problems by providing a framework to highlight the similarities and differences across studies and theories. For instance, we should probably not expect the neural implementation of an episodically originated, sequential, single-input, temporally precise and high complexity prediction (such as turning a corner on a street you walked along yesterday, predicting to see a specific building come into view) to be similar to that of a semantically originated, sequential, multi-input, temporally imprecise and high-complexity prediction (such as walking into a novel bathroom at a restaurant). Moreover, directly testing for these similarities and differences could be key to asserting whether predictive processing principles hold across domains. A parametric manipulation of at least some of these dimensions might further reveal the point at which dimensions break down or reveal new connections between dimensions.

Although we acknowledge that these dimensions are not fully orthogonal to each other, we argue that they allow the characterisation and comparison of the different ways prediction is most frequently used in the cognitive literature (Fig. 1). Moreover, in the light of new evidence, additional dimensions may be needed to fully account for future instantiations of predictions and expectations. Here, we highlight the variety of ways predictions are conceived of and argue that careful consideration of the parameters of prediction is needed to avoid un-warranted generalisations of conclusions across studies, domains, and populations.

Finally, predictions during visual perception do not operate in isolation but have consequences for various cognitive domains. For example, the specificity and mnemonic origins dimensions are most relevant to models which aim to account for the relationship between predictions and declarative memory functions (Henson and Gagnepain, 2010), while arguably all dimensions can influence memory-guided decision making (Shohamy and Adcock, 2010). In addition, predictive processing accounts of motor action planning (e.g., a goalkeeper predicting the trajectory of a football to move and attempt to catch the ball; Ridderinkhof and Brass, 2015) will rely on recursive and temporally precise predictions. In contrast, predictive models of Schizophrenia that include a decreased response to unpredicted rewards (e.g., Katthagen, Kaminski, Heinz, Buchert, & Schlagenhauf, 2020), might concern specific and sequential predictions which are very different from recursive semantic ones. The multidimensional characterisation of predictions presented here can help to guide new research questions that target not-yet-explored combinations of parameters and provide new perspectives on how predictions can help shape future cognition and behaviour.
